# The RSVP Project: Factors Related to Disengagement From Human Immunodeficiency Virus Care Among Persons in San Francisco

**DOI:** 10.2196/publichealth.7325

**Published:** 2017-05-04

**Authors:** Susan Scheer, Miao-Jung Chen, Maree Kay Parisi, Maya Yoshida-Cervantes, Erin Antunez, Viva Delgado, Nicholas J Moss, Kate Buchacz

**Affiliations:** ^1^ HIV Epidemiology Section San Francisco Department of Public Health San Francisco, CA United States; ^2^ Disease Prevention and Control San Francisco Department of Public Health San Francisco, CA United States; ^3^ Division of Communicable Disease Control and Prevention Alameda County Public Health Department Oakland, CA United States; ^4^ Divisions of HIV/AIDS Prevention Centers for Disease Control and Prevention Atlanta, GA United States

**Keywords:** HIV care, HIV care continuum, engagement in HIV care, HIV surveillance, viral suppression, linkage and navigation to care

## Abstract

**Background:**

In the United States, an estimated two-thirds of persons with human immunodeficiency virus (HIV) infection do not achieve viral suppression, including those who have never engaged in HIV care and others who do not stay engaged in care. Persons with an unsuppressed HIV viral load might experience poor clinical outcomes and transmit HIV.

**Objective:**

The goal of the Re-engaging Surveillance-identified Viremic Persons (RSVP) project in San Francisco, CA, was to use routine HIV surveillance databases to identify, contact, interview, and reengage in HIV care persons who appeared to be out of care because their last HIV viral load was unsuppressed. We aimed to interview participants about their HIV care and barriers to reengagement.

**Methods:**

Using routinely collected HIV surveillance data, we identified persons with HIV who were out of care (no HIV viral load and CD4 laboratory reports during the previous 9-15 months) and with their last plasma HIV RNA viral load >200 copies/mL. We interviewed the located persons, at baseline and 3 months later, about whether and why they disengaged from HIV care and the barriers they faced to care reengagement. We offered them assistance with reengaging in HIV care from the San Francisco Department of Public Health linkage and navigation program (LINCS).

**Results:**

Of 282 persons selected, we interviewed 75 (26.6%). Of these, 67 (89%) reported current health insurance coverage, 59 (79%) had ever been prescribed and 45 (60%) were currently taking HIV medications, 59 (79%) had seen an HIV provider in the past year, and 34 (45%) had missed an HIV appointment in the past year. Reasons for not seeing a provider included feeling healthy, using alcohol or drugs, not having enough money or health insurance, and not wanting to take HIV medicines. Services needed to get to an HIV medical care appointment included transportation assistance, stable living situation or housing, sound mental health, and organizational help and reminders about appointments. A total of 52 (69%) accepted a referral to LINCS. Additionally, 64 (85%) of the persons interviewed completed a follow-up interview 3 months later and, of these, 62 (97%) had health insurance coverage and 47 (73%) reported having had an HIV-related care appointment since the baseline interview.

**Conclusions:**

Rather than being truly out of care, most participants reported intermittent HIV care, including recent HIV provider visits and health insurance coverage. Participants also frequently reported barriers to care and unmet needs. Health department assistance with HIV care reengagement was generally acceptable. Understanding why people previously in HIV care disengage from care and what might help them reengage is essential for optimizing HIV clinical and public health outcomes.

## Introduction

In the United States, an estimated two-thirds of persons living with human immunodeficiency virus (HIV) infection do not achieve viral suppression, including both those who have never engaged in HIV care and those who linked to HIV care after diagnosis but did not stay engaged [1-3]. Persons with an unsuppressed HIV viral load might experience poor clinical outcomes and transmit HIV [4] The National HIV/AIDS Strategy for the United States focuses on improving the HIV care continuum, including interventions that link, retain, and reengage persons in HIV care [5,6].

Retention in HIV care has been studied in surveillance registries [2,7-9], observational cohorts [10], health care databases and networks [11-14], and research populations [15,16]. In San Francisco, 19% of persons diagnosed with HIV in 2006-2007 were not adequately retained in care (did not have at least two laboratory measurements reported annually) [17]. Other US jurisdictions have documented reductions in retention after the initial HIV diagnosis and linkage to care, and HIV care disparities in population subgroups [8,18].

The goal of the Re-engaging Surveillance-identified Viremic Persons (RSVP) project in San Francisco was to use routine HIV surveillance databases to identify, contact, interview, and reengage persons living with HIV infection who appeared to be out of care because their last HIV viral load was unsuppressed. We interviewed participants about their HIV care patterns and barriers to reengagement. Understanding why people disengage from HIV care and what would help them reengage is essential for optimizing HIV clinical and public health outcomes.

## Methods

RSVP began on April 20, 2012 for a 12-month period. Our project methods and implementation, including success in locating truly viremic out-of-care persons, have been previously described [19]. Briefly, persons with an unsuppressed plasma HIV RNA viral load (>200 copies/mL) at their last measurement were eligible if they appeared to have disengaged from HIV care because they lacked HIV viral load and CD4 cell count laboratory reports during the 9 to 15 months prior to April 20, 2012 in the San Francisco Department of Public Health Enhanced HIV/AIDS Reporting System. We asked participants to complete a 30-minute interviewer-administered survey and offered them assistance with reengaging in HIV care from the San Francisco Department of Public Health linkage and navigation program (LINCS). Baseline interview questions included demographics, health insurance coverage, HIV testing and care experiences, treatment use, sexual activities, unmet services, and willingness to talk with LINCS. Participants were asked to complete a follow-up interview 3 months later that assessed changes since baseline and care reengagement. RSVP participants could meet with LINCS staff for health insurance assistance, appointments for HIV care reengagement, and referrals to ancillary services. The US Centers for Disease Control and Prevention and the University of California, San Francisco determined this to be a nonresearch activity; therefore, we did not require institutional review board approval.

## Results

### Baseline RSVP Interview

The characteristics of the 75 (26.6%) interviewed participants were broadly representative of the 282 eligible persons [19]. Most of the 75 interviewed participants (median age 45 years) were male (85%), born in the United States (77%), and current San Francisco residents (75%), and identified as gay or homosexual (69%) (Table 1). Participants were racially and ethnically diverse, one-third reported a college degree or higher, the majority were either unemployed or receiving disability benefits, and 1 in 5 reported being homeless or in unstable housing in the previous 12 months. A total of 64 (85%) were sexually active in the past year, and 19 (25%) reported having had condomless anal or vaginal sex with a person of HIV-negative or unknown status. Drug use and binge drinking were common as were symptoms of depression (Table 1).

**Table 1:** Baseline characteristics of interviewed Re-engaging Surveillance-identified Viremic Persons (RSVP) participants, San Francisco, 2012-2013 (n=75).

Characteristics			Median (IQR^a^) or n (%)
**Demographics**			
	**Age (years)**		
		Median (IQR)	45 (37-51)
		<30, n (%)	5 (7)
		30-44, n (%)	31 (41)
		≥45, n (%)	39 (52)
	**Gender, n (%)**		
		Male	64 (85)
		Female	10 (13)
		Transgender	1 (1)
	**Sexual orientation, n (%)**		
		Gay or homosexual	52 (69)
		Straight or heterosexual	12 (16)
		Bisexual	9 (12)
		Questioning	1 (1)
		Queer	1 (1)
	**Country or territory of birth, n (%)**		
		United States	58 (77)
		Puerto Rico	1 (1)
		Mexico	5 (7)
		Other	11 (15)
	**Current city of residence, n (%)**		
		San Francisco	56 (75)
		Other	19 (25)
	**Race/ethnicity, n (%)**		
		Non-Hispanic white	32 (43)
		Non-Hispanic black	20 (27)
		Non-Hispanic Asian/Pacific Islander	2 (3)
		Hispanic/Latino	20 (27)
		Other	1 (1)
	**Education, n (%)**		
		High school, General Equivalency Diploma, or less	22 (29)
		Some technical or college training	30 (40)
		College degree or more	23 (31)
	**Current housing situation, n (%)**		
		Person’s own house or apartment	43 (57)
		Someone else’s house or apartment	16 (21)
		Single room, rented room, motel, single-room occupancy	11 (15)
		All other (shelter, transitional housing, homeless)	5 (7)
	**Current work situation, n (%)**		
		Working full-time or part-time	25 (33)
		Unemployed, laid off, looking for work	25 (33)
		Disabled (receiving disability income)	16 (21)
		Other	3 (4)
		Missing data	6 (8)
	**In jail, detention, or prison in the past 12 months, n (%)**		
		Yes	2 (3)
		No	73 (97)
**Sexual risk, drug use, and other behaviors (past 12 months)**			
	**Had any sex (anal, vaginal, or oral), n (%)**		
		Yes	64 (85)
		No	11 (15)
	**Had anal or vaginal sex without a condom with a person of HIV^b^** **-negative or unknown status, n (%)**		
		Yes	19 (25)
		No	45 (60)
		Not applicable (not sexually active)	11 (15)
	**Injected any nonprescription drugs, n (%)**		
		Yes	18 (24)
		No	55 (73)
		Missing data	2 (3)
	**Used the following drugs (not mutually exclusive), n (%)**		
		Powder cocaine	12 (16)
		Crack cocaine	14 (19)
		Poppers	20 (27)
		Heroin	5 (7)
		Methamphetamine	27 (36)
		Prescription drugs or painkillers without a prescription	16 (21)
	**Binge drinking (≥5, if male, and ≥4, if female, alcoholic drinks in one sitting), n (%)**		
		Daily or weekly	15 (20)
		Monthly or less	21 (28)
		Never	39 (52)
**Health and care utilization**			
	**Has health insurance or other health care coverage, n (%)**		
		Yes	67 (89)
		No	8 (11)
	**Has one place in particular where usually goes for medical care not related to HIV infection, n (%)**		
		Yes	44 (59)
		No	30 (40)
		Missing data	1 (1)
	**Years since first HIV-positive test, n (%)**		
		<5	24 (32)
		5-20	38 (51)
		>20	13 (17)
	**Has seen a provider for HIV medical care in the past 12 months, n (%)**		
		Yes	59 (79)
		No	11 (15)
		Missing data	5 (7)
	**Has had a CD4 cell count or HIV viral load test in the past 12 months, n (%)**		
		Yes	67 (89)
		No	5 (7)
		Missing data/don’t know	3 (4)
	**Ever prescribed HIV medications by a doctor, n (%)**		
		Yes	59 (79)
		No	16 (21)
	**Currently taking any medications to treat HIV infection, n (%)**		
		Yes	45 (60)
		No	30 (40)
	**Missed any HIV medical care appointments in the past 12 months, n (%)**		
		Yes	34 (45)
		No	39 (52)
		Missing data	2 (3)
	**Ever told anyone (other than doctor, nurse, or health care worker) about being HIV-positive, n (%)**		
		Yes	71 (95)
		No	4 (5)
	**Little interest or pleasure in doing things (in the past 2 weeks), n (%)**		
		Yes	34 (45)
		No	41 (55)
	**Feeling down, depressed, or hopeless (in the past 2 weeks), n (%)**		
		Yes	40 (53)
		No	35 (47)
	**Interested in talking with San Francisco Department of Public Health linkage and navigation program** **(LINCS) staff, n (%)**		
		Yes	52 (69)
		No	21 (28)
		Missing	2 (3)

^a^IQR: interquartile range.

^b^HIV: human immunodeficiency virus.

A total of 67 (89%) reported current health insurance or coverage, 59 (79%) had ever been prescribed HIV medications, and 45 (60%) reported current medication use. In the past year, 59 (79%) had seen an HIV provider for medical care, and 67 (89%) reported a CD4 cell count or viral load test. For the 11 (15%) participants who had not seen an HIV provider in the past year, the frequently reported reasons included feeling healthy (n=6), drinking alcohol or using drugs (n=5), not having enough money or health insurance (n=4), and not wanting to take HIV medicines (n=4).

Nearly half (45%) reported missing an HIV medical care appointment in the past year (Table 1). Among 59 participants who reported seeing a provider in the past year, 27 (46%) reported missing an HIV medical care appointment in the past year. These 27 participants volunteered 1 or more of these responses to “What would help you the most to get to HIV medical care appointment within the next 3 months?”: transportation assistance (n=8), a stable living situation or housing (n=3), sound mental health (n=3), help getting organized to track appointments (n=3), and more appointment reminders (phone calls, text messages, email, letters) (n=3).

The 30 (40%) participants who reported not currently taking medications to treat HIV were similar in their characteristics to the overall interviewed population: 24 were men, 25 had current health insurance or coverage, 20 had seen an HIV provider for medical care in the past year, and 23 expressed interest in meeting with LINCS staff. Overall, the most frequently reported services used in the past year were HIV education or information, public benefits support, HIV case management, financial assistance, and spiritual support (Table 2). The most frequently reported needed but not accessed services were dental services, mental health services, financial assistance, transportation assistance, and HIV case management (Table 2).

**Table 2:** Services used, and services that were needed but not used, among the Re-engaging Surveillance-identified Viremic Persons (RSVP) project participants at baseline, San Francisco, 2012-2013 (n=75).

Services in the past 12 months	Used, n (%)	Unmet need^a^: did not use the service but needed it, n (%)
Dental services	23 (31)	35 (67)^b^
Mental health services, including one-to-one counseling	19 (25)	28 (50)^b^
Transportation assistance	19 (25)	26 (46)^b^
Practical support (bills, buddy program, help with cleaning)	12 (16)	25 (40)
Financial assistance	28 (37)^b^	23 (49)^b^
Legal services	13 (17)	22 (35)
Shelter or housing services	8 (11)	21 (31)
HIV^c^ case management services	29 (39)^b^	20 (43)^b^
Drug or alcohol counseling or treatment	13 (17)	18 (29)
Meal or food services	25 (33)	16 (32)
HIV peer group support	15 (20)	16 (27)
Public benefits, including Supplemental Security Income or Social Security Disability Insurance	34 (45)^b^	15 (37)
Spiritual support	27 (36)^b^	14 (29)
Domestic violence services	4 (5)	10 (14)
Education or information about HIV	39 (52)^b^	6 (17)
Interpreter services	0 (0)	1 (1)
Childcare services	1 (1)	0 (0)
Any other HIV-related services	6 (8)	3 (4)

^a^Ordered by highest count; percentage calculated among those who did not use a service in past 12 months.

^b^The top 5 most frequently used services, and the top 5 most frequently needed services (unmet needs).

^c^HIV: human immunodeficiency virus.

Of the participants, 52 (69%) accepted referral to LINCS; their linkage outcomes were previously published [19]. Among the 11 participants who reported not having seen an HIV medical provider in the previous 12 months, 10 (91%) agreed to talk to LINCS staff about reengagement or ancillary services. Of these, 8 (80%) did meet with LINCS staff and, of these, 5 (63%) were already in HIV care and 3 (38%) reengaged in care within 6 months with LINCS assistance.

### Follow-Up Interview

Of the 75 participants, 64 (85%) completed the 3-month follow-up interview. Among the 64, 97% (n=62) had health insurance or coverage, 73% (n=47) reported having “seen a doctor, nurse or other health care provider for HIV medical care” since baseline interview, 81% (n=52) reported having a CD4 cell count test, 77% (n=49) had an HIV viral load test, and 47% (n=30) were prescribed HIV treatment. HIV risk behaviors since the baseline visit included using “any drugs that were not prescribed by a doctor” (n=30, 47%,) and injecting drugs that were not prescribed (n=9, 14%). A total of 33 (52%) reported vaginal or anal sex, including 7 (11%) who reported condomless vaginal or anal sex with an HIV-negative or unknown-status partner.

## Discussion

The RSVP project sought to identify a high-priority population of persons living with HIV who appeared to be out of care and viremic based on HIV surveillance data. However, the majority of interviewed participants reported both having seen an HIV provider in the past 12 months and having health insurance, thus having means and opportunity to access the HIV care needed to achieve viral suppression. The frequent self-reported care engagement was corroborated by HIV surveillance data: over 80% had at least one viral load or CD4 cell count test during the 12 months after they met RSVP project eligibility [19].

Nevertheless, RSVP participants identified personal and structural barriers to HIV care and many unmet needs. Almost half reported missing a scheduled medical appointment in the previous year, and 40% reported no current HIV treatment. Deficits in comprehensive health care and social support included unmet needs for legal services, dental care, transportation assistance, mental health services, financial assistance, and practical support. Notably, about one-fourth of participants reported engaging in condomless sex with negative or unknown HIV-status partners. Given that all RSVP participants had an unsuppressed HIV viral load at their last measurement, the risk of further HIV transmission was possible, indicating that we reached persons who would benefit from assistance remaining in HIV care.

Similar to our findings, a myriad of social, behavioral, and structural factors have previously been found to be correlated with poor retention in care [20,21], including substance and alcohol use, poor mental health, homelessness, and low socioeconomic status [8,10,22]; low care satisfaction, medical establishment distrust, stigma, and lack of social or ancillary support services [15,23]; and early HIV disease or feeling well [8,17]. Patient perceptions of HIV care engagement, including when accessing care only sporadically, may also differ from standard care engagement metrics [24]. The multifaceted barriers to staying engaged in HIV care point to the need for comprehensive, ongoing, innovative, client-centered approaches to support retention (eg, case management, wraparound services, and contingency management). We were unable to analyze specific associations between barriers participants faced and the services they used, and their likelihood of reengaging in care, due to the heterogeneity of barriers and care patterns in our relatively small interviewed population.

In summary, among persons presumed to be out of HIV care, there were self-reported indicators of at least intermittent HIV care, as well as frequently reported barriers to care and unmet needs. As previously described, locating persons who are truly out of HIV care is difficult [19]. Nevertheless, surveillance-based public health efforts support the HIV care continuum, as most RSVP project participants returned for a follow-up interview, seemed willing to engage with health department staff, and accepted assistance with ancillary services and HIV care reengagement, a prerequisite for ongoing HIV treatment and viral suppression.

## Abbreviations

HIV: human immunodeficiency virus
LINCS: San Francisco Department of Public Health linkage and navigation program
RSVP: Re-engaging Surveillance-identified Viremic Persons
